# Supplementary material for: Impacts on tundra vegetation from heavy metal-enriched fugitive dust on National Park Service lands along the Red Dog Mine haul road, Alaska
Source: PLoS One. 2022 Jun 13;17(6):e0269801. doi: 10.1371/journal.pone.0269801 (PMC9191729; doi:10.1371/journal.pone.0269801)
Supplement: S1 Fig — Prediction points inside of the AIDEA industrial easement are shown in blue, while those outside the easement appear in pink. The 95% confidence interval is shown in grey. A log LSR of 1.5 is approximately 31.6, or 75% of the mean LSR of the highest stratum. (PDF) [file pone.0269801.s001.pdf]

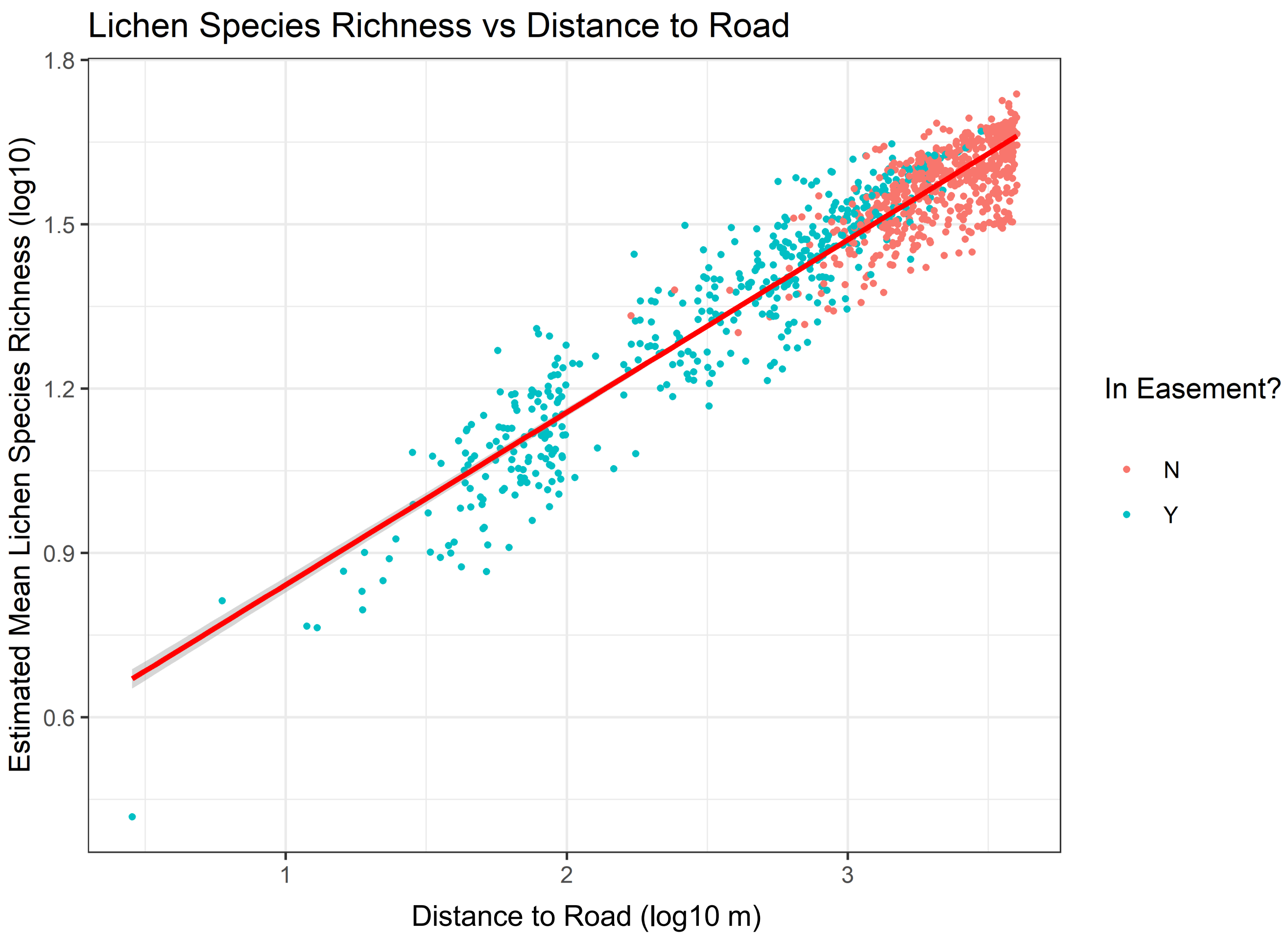

S1 Fig. Estimated mean lichen species richness vs. distance to the DMTS haul road in CAKR on a log-log scale from a Bayesian posterior predictions model, classed by easement status. Prediction points inside of the NANA/AIDEA industrial easement are shown in blue, while those outside the easement appear in pink. The 95% confidence interval is shown in grey. A log LSR of 1.5 is approximately 31.6, or 75% of the mean LSR of the highest stratum.
