# Supplementary material for: Impacts on tundra vegetation from heavy metal-enriched fugitive dust on National Park Service lands along the Red Dog Mine haul road, Alaska
Source: PLoS One. 2022 Jun 13;17(6):e0269801. doi: 10.1371/journal.pone.0269801 (PMC9191729; doi:10.1371/journal.pone.0269801)

S2 Fig. Elemental concentrations in *Hylocomium splendens* moss (mg/kg) along the DMTS haul road in CAKR graphed with best-fit non-linear curves for each element.

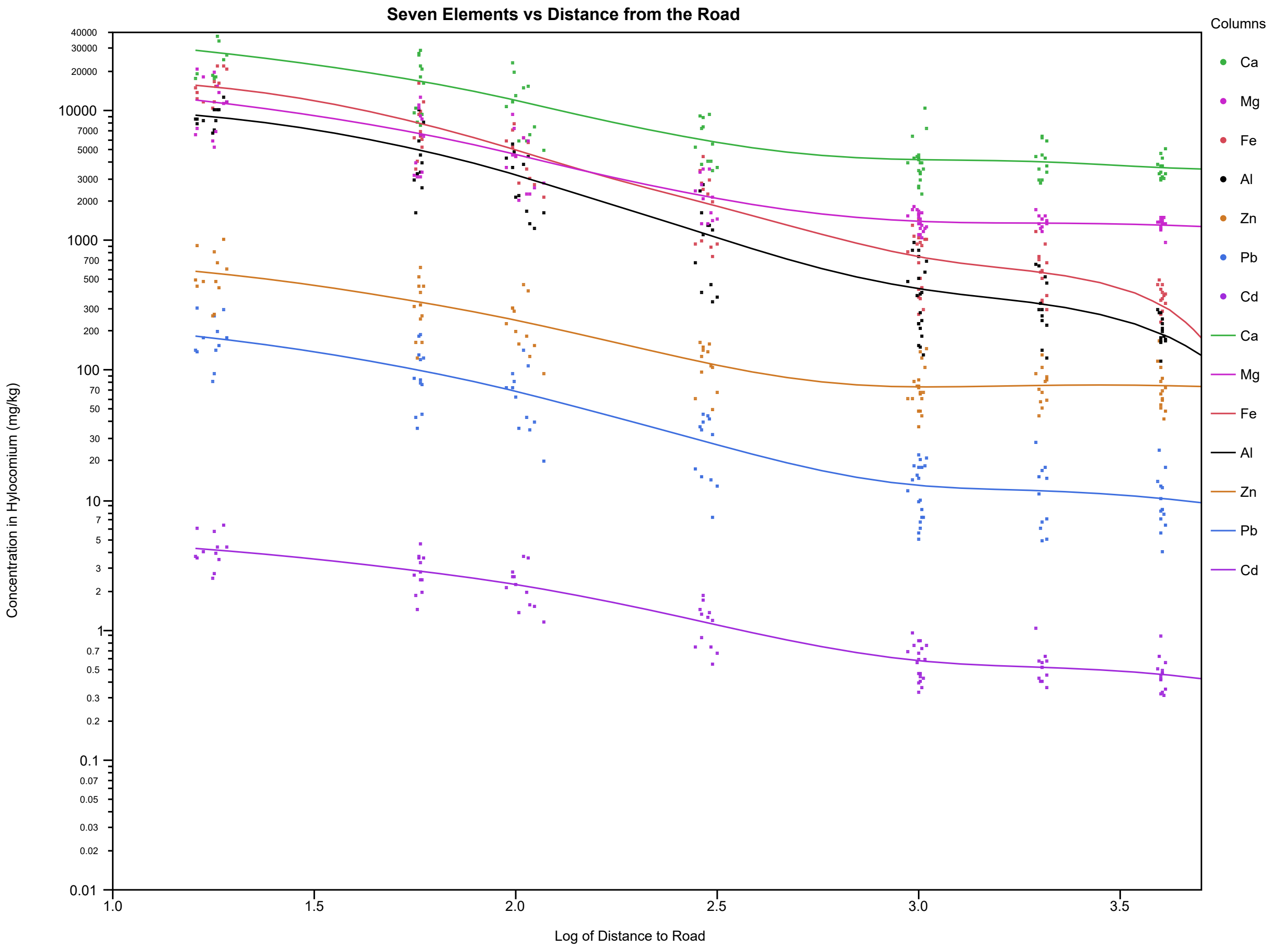

Supplement: S2 Fig — (PDF) [file pone.0269801.s002.pdf]
