# Supplementary material for: Impacts on tundra vegetation from heavy metal-enriched fugitive dust on National Park Service lands along the Red Dog Mine haul road, Alaska
Source: PLoS One. 2022 Jun 13;17(6):e0269801. doi: 10.1371/journal.pone.0269801 (PMC9191729; doi:10.1371/journal.pone.0269801)
Supplement: S1 Table — (PDF) [file pone.0269801.s003.pdf]

S1 Table. Percent frequency of macrolichen species found on vegetation plots by distance class from the DMTS haul road in CAKR.

Taxon	Total Percent Frequency	% Freq 10m	% Freq 50m	% Freq 100m	% Freq 300m	% Freq 1000m	% Freq 2000m	% Freq 4000m
Alectoria nigricans	6	-	-	8	-	10	8	-
Alectoria ochroleuca	7	8	-	-	8	10	8	14
Asahinea chrysantha	16	-	-	-	8	30	17	43
Bryocaulon divergens	18	-	-	-	25	25	25	43
Bryoria nitidula	1	-	-	-	-	5	-	-
Bryoria simplicior	7	-	-	-	-	15	17	-
Cetraria sp.	1	-	-	-	-	-	-	-
Cetraria fastigiata	1	-	-	-	-	-	-	-
Cetraria inermis	30	-	8	25	58	30	33	29
Cetraria islandica	55	-	33	50	50	70	75	57
Cetraria laevigata	72	8	67	50	75	100	92	71
Cetraria sepincola	75	8	5-	58	92	100	92	93
Cetrelia alaskana	3	-	-	-	-	-	17	7
Cladonia sp.	31	8	17	17	42	45	42	43
Cladonia albonigra	4	-	8	-	-	-	17	7
Cladonia amaurocraea	87	42	75	83	100	95	100	93
Cladonia arbuscula	82	17	67	92	92	95	92	93
Cladonia bacilliformis	32	-	-	17	25	75	25	21
Cladonia bellidiflora	11	-	-	-	25	20	8	21
Cladonia carneola	33	-	-	-	-	35	75	79
Cladonia cenotea	14	-	8	8	8	20	8	14
Cladonia cervicornis	1	-	-	-	-	-	-	-
Cladonia chlorophaea	17	-	17	25	25	15	33	21
Cladonia coccifera	32	-	-	-	33	55	58	79
Cladonia coniocraea	21	-	8	-	25	15	58	57
Cladonia cornuta	69	8	33	67	92	75	92	100
Cladonia crispata	20	-	-	8	25	25	25	43
Cladonia cyanipes	26	-	8	17	25	30	58	29

Cladonia decorticata	7	-	-	-	-	5	8	36
Cladonia deformis	40	8	8	25	50	75	67	50
Cladonia digitata	5	-	8	-	-	10	8	-
Cladonia ecmocyna	5	-	-	-	8	5	-	21
Cladonia fimbriata	62	8	25	67	50	65	92	100
Cladonia gracilis ssp. elongata	49	17	33	25	42	70	83	64
Cladonia gracilis ssp. turbinata	21	-	33	33	8	10	58	21
Cladonia macroceras	1	-	-	-	-	-	-	7
Cladonia macrophyllodes	1	-	-	-	-	-	8	-
Cladonia maxima	74	17	58	83	83	85	83	71
Cladonia merochlorophaea	23	-	-	17	50	10	33	21
Cladonia metacorallifera	5	-	8	-	-	-	-	21
Cladonia ochrochlora	48	8	25	50	42	60	58	79
Cladonia phyllophora	47	17	17	50	58	55	67	79
Cladonia pleurota	24	-	8	-	33	35	33	57
Cladonia pocillum	3	-	-	-	8	5	8	-
Cladonia pyxidata	28	-	8	17	42	40	33	43
Cladonia rangiferina	75	17	50	75	75	95	100	86
Cladonia scabriuscula	17	-	8	-	8	20	25	36
Cladonia singularis	2	-	-	-	8	5	-	-
Cladonia squamosa	43	-	17	25	33	50	83	86
Cladonia stellaris	2	-	-	8	8	-	-	-
Cladonia stricta	5	-	-	-	-	10	8	7
Cladonia stygia	75	17	75	58	83	80	92	86
Cladonia subfurcata	18	-	-	-	-	10	50	50
Cladonia sulphurina	52	-	-	50	50	70	75	86
Cladonia transcendens	12	-	-	-	-	10	42	43
Cladonia uncialis	56	-	42	33	58	85	75	86
Cladonia wainioi	1	-	-	-	-	5	-	-
Dactylina arctica	22	8	-	17	42	30	42	21
Dactylina ramulosa	3	-	-	-	-	-	8	14
Flavocetraria cucullata	88	33	83	83	100	100	100	93
Flavocetraria nivalis	22	8	-	8	-	55	25	36
Hypogymnia sp.	1	-	-	-	-	-	-	7
Hypogymnia austerodes	2	-	-	-	-	-	8	-

Hypogymnia physodes	22	-	17	8	25	45	8	29
Hypogymnia subobscura	19	8	-	8	17	30	25	29
Icmadophila ericetorum	2	-	-	-	-	-	-	14
Lasallia pensylvanica	1	-	-	-	-	-	-	7
Lobaria linita	7	-	-	8	-	10	8	7
Lobaria pseudopulmonaria	25	-	17	17	17	20	58	43
Lobaria scrobiculata	1	-	-	-	-	-	8	-
Masonhalea richardsonii	3	-	-	-	-	5	8	7
Melanelia olivacea	2	-	-	-	-	-	17	-
Melanelia septentrionalis	70	8	50	67	83	80	83	86
Nephroma sp.	1	-	-	8	-	-	-	-
Nephroma arcticum	23	-	17	33	50	15	42	29
Nephroma expallidum	25	8	17	17	8	35	50	29
Ochrolechia frigida	11	-	-	-	8	15	17	29
Pannaria pezzizoides	7	-	-	-	-	15	8	7
Parmelia sp.	1	-	-	-	-	5	-	-
Parmelia omphalodes	17	-	8	-	17	30	25	43
Parmelia saxatilis	3	-	-	-	-	-	8	14
Parmelia sulcata	38	8	17	50	17	60	42	36
Parmeliopsis ambigua	32	-	-	-	42	45	58	50
Parmeliopsis hyperopta	50	8	17	25	50	75	83	36
Peltigera sp.	6	17	-	17	-	-	-	7
Peltigera aphthosa	69	17	50	58	75	75	92	86
Peltigera canina	6	-	8	-	-	5	25	-
Peltigera didactyla	13	8	17	25	25	20	8	-
Peltigera kristinssonii	7	-	-	-	-	10	17	7
Peltigera leucophlebia	19	8	8	-	-	5	58	43
Peltigera malacea	16	8	17	8	8	15	33	29
Peltigera membranacea	4	8	-	-	8	-	-	-
Peltigera polydactylon	46	-	17	17	50	55	75	79
Peltigera rufescens	7	8	8	8	8	10	8	-
Peltigera scabrosa	52	17	42	33	50	55	83	71
Pertusaria dactylina	5	-	-	-	-	10	17	7
Physcia sp.	1	-	-	-	-	-	-	-
Physcia aipolia	3	-	-	-	-	5	8	7

Physcia caesia	1	-	-	-	-	-	-	-
Protopannaria pezizoides	1	-	-	-	8	-	-	-
Psoroma hypnorum	22	-	-	8	8	25	42	29
Ramalina roesleri	2	-	-	-	8	-	-	-
Solorina bispora	1	-	-	-	-	5	-	-
Solorina saccata	1	-	-	-	-	5	-	-
Sphaerophorus fragilis	6	-	-	-	8	25	-	-
Sphaerophorus globosus	27	-	17	-	17	50	42	57
Stereocaulon sp.	2	-	-	-	-	-	-	14
Stereocaulon grande	1	-	-	-	-	-	-	-
Stereocaulon paschale	13	-	-	8	-	30	25	21
Stereocaulon tomentosum	4	-	-	-	-	-	8	21
Thamnolia subuliformis/vermicularis	81	67	100	92	83	85	100	93
Vulpicida pinastri	63	17	25	42	50	85	83	86
Unknown	2	-	-	-	-	-	-	14
