# Supplementary material for: Impacts on tundra vegetation from heavy metal-enriched fugitive dust on National Park Service lands along the Red Dog Mine haul road, Alaska
Source: PLoS One. 2022 Jun 13;17(6):e0269801. doi: 10.1371/journal.pone.0269801 (PMC9191729; doi:10.1371/journal.pone.0269801)
Supplement: S2 Table — (PDF) [file pone.0269801.s004.pdf]

S2 Table. Mean percent lichen cover in plots within different distance classes from the DMTS haul road in CAKR.

Distance Class (m)	N	Mean % Cover Lichens	SE	Tukey-Kramer Significance Group
10	12	0.6	1.2	C
50	12	2.7	1.2	BC
100	12	2.7	1.2	BC
300	12	6.7	1.2	AB
1000	20	9.3	1.0	A
2000	12	5.3	1.2	ABC
4000	14	8.5	1.1	A
