# Supplementary material for: Impacts on tundra vegetation from heavy metal-enriched fugitive dust on National Park Service lands along the Red Dog Mine haul road, Alaska
Source: PLoS One. 2022 Jun 13;17(6):e0269801. doi: 10.1371/journal.pone.0269801 (PMC9191729; doi:10.1371/journal.pone.0269801)
Supplement: S3 Table — Excluded were reference plots, Kotzebue plots and 3 plots for which there were several missing values. Hyl spl: Hylocomium splendens moss. Fla cuc: Flavocetraria cucullata lichen. Cet lae: Cetraria laevigata lichen. (PDF) [file pone.0269801.s005.pdf]

S3 Table. Correlation matrix (r) of elemental concentrations in *Hylocomium splendens* and a subset of biological variables from 91 plots along the DMTS haul road, Cape Krusenstern National Monument, Alaska. Excluded were reference plots, Kotzebue plots and 3 plots for which there were several missing values. Hyl spl: *Hylocomium splendens moss. Fla cuc: Flavocetraria cucullata* lichen. Cet lae: *Cetraria laevigata* lichen.

	**Log Dist Road**	**LSR**	**Log Cd 2001**	**Log Pb 2001**	**Log Zn 2001**	**Log Al**	**Log Ca**	**Log Cd**	**Log Cr**	**Log Cu**	**Log Fe**	**Log Mg**	**Log Mn**	**Log Na**	**Log Ni**	**Log P**	**Log Pb**	**Log Zn**	**Log Total S**	**Lot Total N**	**Log Lichen Cover**	**% Hyl spl midrib black**	**Height *Fla cuc* ***	**Height *Cet lae* ****
**Log Distance to Road**	1	0.82	-0.91	-0.93	-0.92	-0.93	-0.87	-0.91	-0.92	-0.89	-0.94	-0.88	0.54	-0.74	-0.92	0.67	-0.89	-0.85	-0.79	0.77	0.71	-0.75	0.29	0.37
**LSR**	0.82	1	-0.72	-0.75	-0.74	-0.83	-0.81	-0.83	-0.82	-0.82	-0.83	-0.82	0.45	-0.70	-0.82	0.61	-0.83	-0.79	-0.72	0.64	0.77	-0.68	-0.01	0.33
**Log Cd 2001**	-0.91	-0.72	1	0.99	0.99	0.87	0.79	0.88	0.88	0.78	0.89	0.77	-0.59	0.69	0.84	-0.58	0.86	0.81	0.70	-0.62	-0.54	0.60	-0.36	-0.29
**Log Pb 2001**	-0.93	-0.75	0.99	1	1.00	0.88	0.81	0.90	0.90	0.80	0.90	0.80	-0.59	0.69	0.85	-0.60	0.88	0.83	0.70	-0.64	-0.57	0.61	-0.36	-0.32
**Log Zn 2001**	-0.92	-0.74	0.99	1.00	1	0.88	0.82	0.90	0.89	0.80	0.90	0.80	-0.59	0.70	0.85	-0.59	0.88	0.84	0.71	-0.62	-0.57	0.61	-0.39	-0.32
**Log Al**	-0.93	-0.83	0.87	0.88	0.88	1	0.91	0.96	0.99	0.93	0.99	0.91	-0.57	0.80	0.96	-0.70	0.96	0.91	0.82	-0.75	-0.72	0.72	-0.29	-0.30
**Log Ca**	-0.87	-0.81	0.79	0.81	0.82	0.91	1	0.93	0.89	0.92	0.92	0.96	-0.60	0.79	0.91	-0.67	0.94	0.93	0.88	-0.72	-0.79	0.77	-0.40	-0.25
**Log Cd**	-0.91	-0.83	0.88	0.90	0.90	0.96	0.93	1	0.95	0.94	0.97	0.93	-0.54	0.78	0.95	-0.68	0.98	0.95	0.85	-0.72	-0.72	0.74	-0.38	-0.31
**Log Cr**	-0.92	-0.82	0.88	0.90	0.89	0.99	0.89	0.95	1	0.89	0.98	0.89	-0.59	0.77	0.94	-0.66	0.94	0.88	0.77	-0.71	-0.66	0.67	-0.27	-0.30
**Log Cu**	-0.89	-0.82	0.78	0.80	0.80	0.93	0.92	0.94	0.89	1	0.94	0.94	-0.41	0.79	0.94	-0.64	0.94	0.96	0.94	-0.74	-0.80	0.83	-0.41	-0.29
**Log Fe**	-0.94	-0.83	0.89	0.90	0.90	0.99	0.92	0.97	0.98	0.94	1	0.92	-0.58	0.79	0.97	-0.71	0.96	0.92	0.83	-0.76	-0.73	0.74	-0.31	-0.29
**Log Mg**	-0.88	-0.82	0.77	0.80	0.80	0.91	0.96	0.93	0.89	0.94	0.92	1	-0.49	0.76	0.92	-0.68	0.92	0.91	0.86	-0.76	-0.80	0.79	-0.34	-0.26
**Log Mn**	0.54	0.45	-0.59	-0.59	-0.59	-0.57	-0.60	-0.54	-0.59	-0.41	-0.58	-0.49	1	-0.50	-0.51	0.51	-0.54	-0.47	-0.38	0.41	0.32	-0.33	0.15	0.16
**Log Na**	-0.74	-0.70	0.69	0.69	0.70	0.80	0.79	0.78	0.77	0.79	0.79	0.76	-0.50	1	0.82	-0.58	0.78	0.79	0.74	-0.68	-0.65	0.66	-0.39	-0.22
**Log Ni**	-0.92	-0.82	0.84	0.85	0.85	0.96	0.91	0.95	0.94	0.94	0.97	0.92	-0.51	0.82	1	-0.68	0.93	0.92	0.85	-0.76	-0.74	0.75	-0.38	-0.38
**Log P**	0.67	0.61	-0.58	-0.60	-0.59	-0.70	-0.67	-0.68	-0.66	-0.64	-0.71	-0.68	0.51	-0.58	-0.68	1	-0.68	-0.63	-0.58	0.84	0.59	-0.55	0.09	0.07
**Log Pb**	-0.89	-0.83	0.86	0.88	0.88	0.96	0.94	0.98	0.94	0.94	0.96	0.92	-0.54	0.78	0.93	-0.68	1	0.96	0.85	-0.71	-0.73	0.75	-0.36	-0.24
**Log Zn**	-0.85	-0.79	0.81	0.83	0.84	0.91	0.93	0.95	0.88	0.96	0.92	0.91	-0.47	0.79	0.92	-0.63	0.96	1	0.91	-0.68	-0.77	0.76	-0.51	-0.28
**Log Total S**	-0.79	-0.72	0.70	0.70	0.71	0.82	0.88	0.85	0.77	0.94	0.83	0.86	-0.38	0.74	0.85	-0.58	0.85	0.91	1	-0.67	-0.80	0.82	-0.50	-0.33
**Lot Total N**	0.77	0.64	-0.62	-0.64	-0.62	-0.75	-0.72	-0.72	-0.71	-0.74	-0.76	-0.76	0.41	-0.68	-0.76	0.84	-0.71	-0.68	-0.67	1	0.68	-0.67	0.24	0.23
**Log Lichen Cover**	0.71	0.77	-0.54	-0.57	-0.57	-0.72	-0.79	-0.72	-0.66	-0.80	-0.73	-0.80	0.32	-0.65	-0.74	0.59	-0.73	-0.77	-0.80	0.68	1	-0.79	0.37	0.29
**% Hyl spl midrib blackening**	-0.75	-0.68	0.60	0.61	0.61	0.72	0.77	0.74	0.67	0.83	0.74	0.79	-0.33	0.66	0.75	-0.55	0.75	0.76	0.82	-0.67	-0.79	1	-0.20	-0.27
**Height *Fla cuc* ***	0.59	0.17	-0.58	-0.59	-0.58	-0.47	-0.50	-0.57	-0.47	-0.47	-0.50	-0.49	0.38	-0.46	-0.56	0.24	-0.52	-0.60	-0.46	0.40	0.47	-0.36	1	0.43
**Height *Cet lae* ****	0.58	0.09	-0.52	-0.55	-0.49	-0.31	-0.24	-0.48	-0.36	-0.19	-0.35	-0.26	0.41	-0.26	-0.39	0.12	-0.47	-0.37	-0.16	0.40	0.38	-0.31	0.43	1

Unless starred, 16 plots were omitted due to missing or excluded values.

* 20 rows excluded, plots analyzed with Reference Plots included

**39 rows excluded, plots analyzed with Reference Plots included
