# Supplementary material for: Impacts on tundra vegetation from heavy metal-enriched fugitive dust on National Park Service lands along the Red Dog Mine haul road, Alaska
Source: PLoS One. 2022 Jun 13;17(6):e0269801. doi: 10.1371/journal.pone.0269801 (PMC9191729; doi:10.1371/journal.pone.0269801)
Supplement: S4 Table — Species are sorted by Axis 1 r scores. (PDF) [file pone.0269801.s006.pdf]

S4 Table. Lichen species Spearman correlations (r) with NMS ordination Axis 1 and Indicator Species Analysis p values comparing two groups: < 1000 m and 1000–4000 m from the DMTS haul road in CAKR. Species are sorted by Axis 1 r scores.

Lichen Species	r Axis 1	Group Association	ISA p value
Cladonia cornuta	0.671	≥ 1000 m	0.0002
Cetraria sepincola	0.636	≥ 1000 m	0.0002
Cetraria pinastri	0.593	≥ 1000 m	0.0004
Cladina arbuscula	0.583	≥ 1000 m	0.0016
Cladonia fimbriata	0.581	≥ 1000 m	0.0002
Cetraria laevigata	0.573	≥ 1000 m	0.0002
Cetraria cucullata	0.555	≥ 1000 m	0.0016
Cladina rangiferina	0.534	≥ 1000 m	0.0002
Cladonia sulphurina	0.523	≥ 1000 m	0.0002
Cladonia amaurocraea	0.516	≥ 1000 m	0.0046
Cladonia uncialis	0.515	≥ 1000 m	0.0002
Cladonia maxima	0.503	≥ 1000 m	0.1178
Cladonia squamosa	0.498	≥ 1000 m	0.0002
Melanelia septentrionalis	0.484	≥ 1000 m	0.0048
Cladonia gracilis ssp. elongata	0.478	≥ 1000 m	0.0002
Cladonia coccifera	0.473	≥ 1000 m	0.0002
Peltigera polydactylon	0.458	≥ 1000 m	0.0002
Cladonia deformis	0.455	≥ 1000 m	0.0006
Cladina stygia	0.453	≥ 1000 m	0.0064
Parmeliopsis hyperopta	0.437	≥ 1000 m	0.0002
Cladonia ochrochlora	0.42	≥ 1000 m	0.0034
Peltigera aphthosa	0.42	≥ 1000 m	0.0012
Cladonia carneola	0.417	≥ 1000 m	0.0002
Cladonia phyllophora	0.414	≥ 1000 m	0.011
Cetraria islandica	0.413	≥ 1000 m	0.0096
Parmeliopsis ambigua	0.397	≥ 1000 m	0.0002
Peltigera scabrosa	0.366	≥ 1000 m	0.0074
Cladonia pleurota	0.36	≥ 1000 m	0.0012
Cladonia pyxidata	0.36	≥ 1000 m	0.0262
Sphaerophorus globosus	0.355	≥ 1000 m	0.0002
Cladonia bacilliformis	0.338	≥ 1000 m	0.0006
Cladonia coniocraea	0.333	≥ 1000 m	0.0024
Hypogymnia physodes	0.326	≥ 1000 m	0.0756
Parmelia omphalodes	0.325	≥ 1000 m	0.0022
Parmelia sulcata	0.319	≥ 1000 m	0.023
Bryocaulon divergens	0.318	≥ 1000 m	0.0074
Lobaria pseudopulmonaria	0.306	≥ 1000 m	0.0124
Cladonia crispata	0.305	≥ 1000 m	0.0132

Cetraria inermis	0.303	≥ 1000 m	0.6471
Cetraria nivalis	0.301	≥ 1000 m	0.0004
Asahinea chrysantha	0.3	≥ 1000 m	0.0004
Psoroma hypnorum	0.299	≥ 1000 m	0.0026
Hypogymnia subobscura	0.295	≥ 1000 m	0.0292
Cladonia cyanipes	0.293	≥ 1000 m	0.0162
Cladonia transcendens	0.283	≥ 1000 m	0.0002
Cladonia subfurcata	0.281	≥ 1000 m	0.0002
Dactylina arctica	0.271	≥ 1000 m	0.2238
Stereocaulon paschale	0.247	≥ 1000 m	0.0016
Cladonia	0.246	≥ 1000 m	0.036
Cladonia bellidiflora	0.243	≥ 1000 m	0.1992
Cladonia merochlorophaea	0.238	≥ 1000 m	1
Peltigera leucophlebia	0.221	≥ 1000 m	0.0024
Ochrolechia frigida	0.215	≥ 1000 m	0.0136
Cladonia scabriuscula	0.212	≥ 1000 m	0.0076
Cladonia chlorophaea	0.207	≥ 1000 m	0.7926
Cladonia decorticata	0.207	≥ 1000 m	0.0136
Nephroma expallidum	0.193	≥ 1000 m	0.0146
Cladonia stricta	0.18	≥ 1000 m	0.1186
Stereocaulon tomentosum	0.178	≥ 1000 m	0.1222
Peltigera malacea	0.175	≥ 1000 m	0.1754
Alectoria ochroleuca	0.171	≥ 1000 m	0.4193
Pannaria pezzizoides	0.171	≥ 1000 m	0.0594
Cladonia cenotea	0.169	≥ 1000 m	0.3097
Cladonia ecmocyna	0.169	≥ 1000 m	0.2947
Sphaerophorus fragilis	0.169	≥ 1000 m	0.1502
Pertusaria dactylina	0.165	≥ 1000 m	0.0506
Cladonia metacorallifera	0.148	≥ 1000 m	0.6179
Cladonia pocillum	0.145	≥ 1000 m	1
unknown	0.145	≥ 1000 m	0.4985
Bryoria simplicior	0.143	≥ 1000 m	0.0488
Dactylina ramulosa	0.14	≥ 1000 m	0.2326
Cladonia digitata	0.137	≥ 1000 m	0.6211
Physcia aipolia	0.13	≥ 1000 m	0.2603
Lobaria linita	0.129	≥ 1000 m	0.3579
Peltigera kristinssonii	0.127	≥ 1000 m	0.0548
Cladonia albonigra	0.121	≥ 1000 m	0.6263
Parmelia saxatilis	0.119	≥ 1000 m	0.2348
Cetrelia alaskana	0.118	≥ 1000 m	0.2428
Alectoria nigricans	0.117	≥ 1000 m	0.6241
Masonhalea richardsonii	0.112	≥ 1000 m	0.2408
Cladonia gracilis ssp. *turbinata*	0.109	≥ 1000 m	0.4913
Nephroma arcticum	0.107	10 - 300 m	0.5589

Cladonia singularis	0.101	10 - 300 m	1
Peltigera canina	0.088	≥ 1000 m	0.3585
Melanelia olivacea	0.067	≥ 1000 m	0.4969
Icmadophila ericetorum	0.065	≥ 1000 m	0.4905
Stereocaulon spp.	0.056	≥ 1000 m	0.4895
Peltigera membranacea	0.041	10 - 300 m	0.237
Thamnolia subuliformis	0.02	10 - 300 m	0.997
Cladina stellaris	-0.016	10 - 300 m	0.2322
Peltigera didactyla	-0.016	10 - 300 m	0.248
Peltigera rufescens	-0.08	10 - 300 m	0.7157
Peltigera sp.	-0.108	10 - 300 m	0.3373
