# Supplementary material for: Impacts on tundra vegetation from heavy metal-enriched fugitive dust on National Park Service lands along the Red Dog Mine haul road, Alaska
Source: PLoS One. 2022 Jun 13;17(6):e0269801. doi: 10.1371/journal.pone.0269801 (PMC9191729; doi:10.1371/journal.pone.0269801)
Supplement: S1 File — (PDF) [file pone.0269801.s007.pdf]

S1 File. Lichen species richness vs. distance to the Dalton Highway in data from Auerbach et al. [(26)] on a log-log scale.

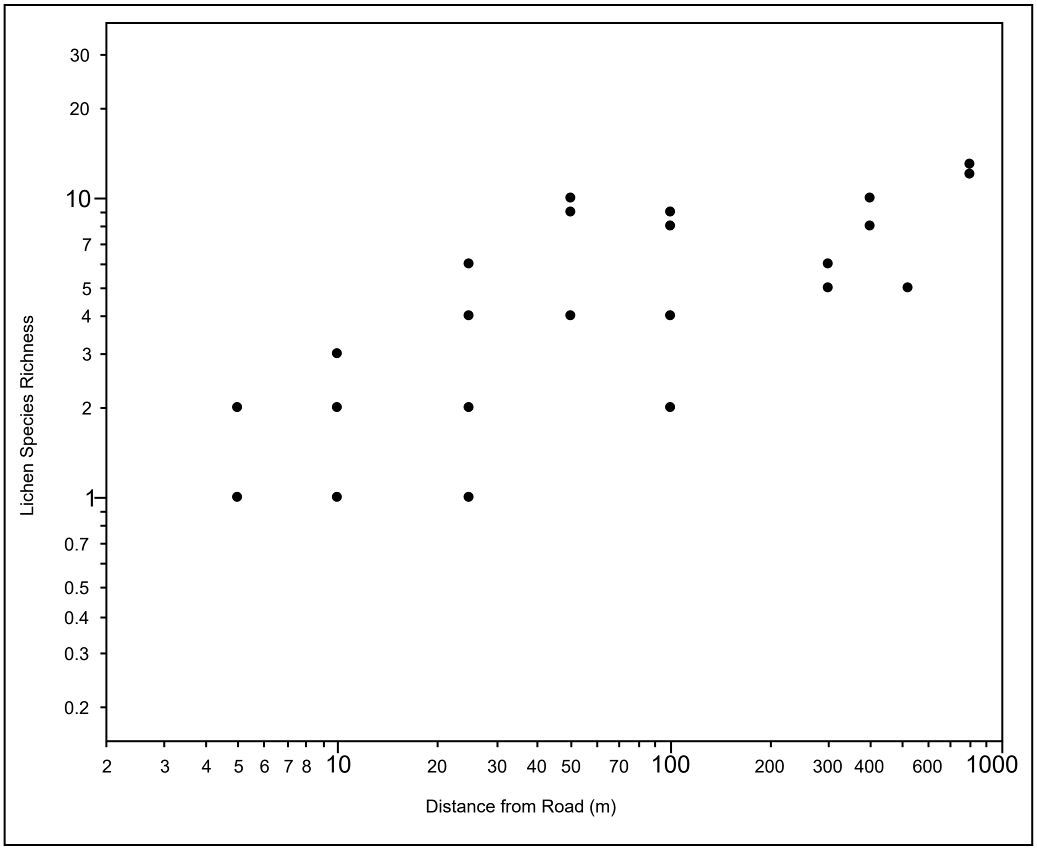

At each the Toolik and the Sagawon sites, there was a strong positive correlation between $\log_{10}$ distance to the road and $\log_{10}$LSR (r^2=0.77 and 0.75, respectively with a grouped r^2=0.55 combining the two sites). The effect of distance from the road on LSR was tested in ANOVA for each site independently and for the pooled data. In each case the 400m, 800m and 50m distance class grouped together as significantly higher ($p<0.001$ in each instance) than the closer distance classes (2 to 20 m), suggesting that the zone of maximum effect of crustal element road dust on LSR was <50m. This zone of influence on lichen biomass, which is closely related to lichen cover, showed only a weak correlation with the log of distance to the road at each site (r^2 = 0.08 and 0.28 respectively).
