# Supplementary material for: Impacts on tundra vegetation from heavy metal-enriched fugitive dust on National Park Service lands along the Red Dog Mine haul road, Alaska
Source: PLoS One. 2022 Jun 13;17(6):e0269801. doi: 10.1371/journal.pone.0269801 (PMC9191729; doi:10.1371/journal.pone.0269801)
Supplement: S2 File — (PDF) [file pone.0269801.s008.pdf]

S2 File. Modeled LSR in current study vs. modeled Zn from Neitlich et al. [17] on a log-log scale.

Table 1. Mean modeled Zn values and standard deviation from Neitlich et al. (2017) at three spatial points of high LSR along the DMTS haul road in CAKR. Ten spatial modeling grid points bracketing the target LSR value were selected for three estimates of modeled Zn concentration. Selected LSR targets were: 1) the mean LSR of the highest stratum (3000-4000m), 2) the ≥ 95% of the mean LSR of the highest stratum used in mapping, and 3) the 3000 m distance representing the beginning of the stratum with highest LSR in stratum-based analysis.

LSR Targets	n	LSR	Mean Zn	SD Zn
Mean LSR of highest stratum (MHS)	10	41.9	62.2	15.5
≥ 95% MHS	10	39.8	58.3	17.1
3000 m (+/- 25 m) distance from DMTS	10	39.2	69.7	21.2

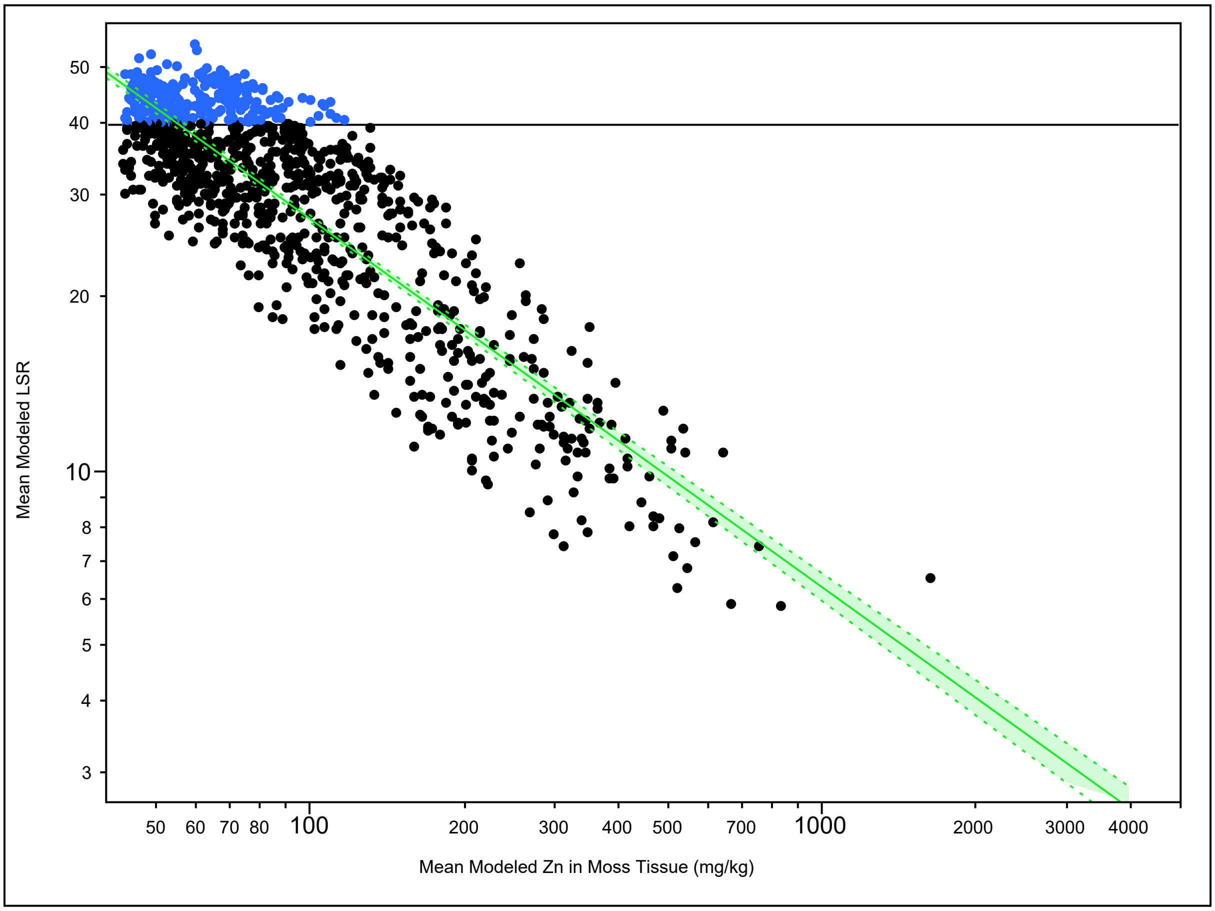

Fig 1. Mean modeled LSR vs. mean modeled Zn in Hylocomium tissue from Neitlich et al. [17] **2017.** Points along the DMTS haul road in CAKR were plotted on a $\log_{10}$-$\log_{10}$ scale. Modeling was based on data collected at the same field points and using the same posterior

prediction grid. Dots at LSR ≥ 39.8 (or 95% of the MHS) are shown in blue. In linear regression, $r^2 = 0.76$, $p < 0.001$.
