# Supplementary material for: Impacts on tundra vegetation from heavy metal-enriched fugitive dust on National Park Service lands along the Red Dog Mine haul road, Alaska
Source: PLoS One. 2022 Jun 13;17(6):e0269801. doi: 10.1371/journal.pone.0269801 (PMC9191729; doi:10.1371/journal.pone.0269801)
Supplement: Attachment — Submitted filename: Comments_to_authors.pdf [file pone.0269801.s009.pdf]

Summary of Research

This manuscript expands upon existing work to assess the effect of fugitive dust on lichen community richness. It expands upon previously published work by focusing primarily on lichen communities and corroborating a spatial model with past results.

The study area, target organisms, and management challenge are highly relevant. The study design provides a good attempt to answer research questions. Data is handled thoroughly and fairly with appropriate statistical methods and species community treatments. The authors place appropriate bounds on their analyses and how it may be applied. They do an excellent job placing their work into context of other similar studies and highlight how this study area, although small, is an excellent case example that allows an interpretation approach near "experimental".

To the best of my knowledge and experience, I recommend this manuscript for acceptance to PLosOne with minor revisions.

In the attached file, I've included general comments requiring standardization throughout parts of the manuscript and specific comments relevant only to one or few lines. I am encouraged by the inclusion of background on this area (i.e., the land ownership and private easement issues) that sometime are omitted in scientific studies even if those issues are what instigated the funding of the research.

General Comments

- Search for references of "the Monument" and standardize capitalization
- Standardize use of metric or miles throughout or include both
- Standardize use of "haul road", perhaps when first introducing the DMTS more formal name state "hereafter referred to as…"
- Standardize use of elemental names and abbreviations and abbreviation definitions throughout
- Consider the use of subheadings in the Introduction if permitted by journal to facilitate comprehensive of the multiple issues (i.e., land ownership, history of deposition, use of nonvascular plants as monitors)
- Nonconventional referencing to citation 17, the preceding study in Plos One, in the Methods and Abstract. Perhaps make more explicit statement of how this article is a continuation/extension of that study?
- Because the reference plots were later omitted, I think the authors can take out any reference to them in methods or results. Given the number of plots, I don't see a real need for even stating that the authors did them even though it was a noble idea. Alternatively, since they are included in the discussion, make clear in the results why they still retain value in environmental comparison, even if they don't for diversity comparison.

Specific Comments

Line 33- end with "within CAKR" instead of "in the monument"- monument is a bit ambiguous

Line 44-47- confusing sentence expressing both negative and positive correlations

Line 48-49- Wow, nicely put. It is easy to envision this impact.

Line 67- the first reference of the NPS acronym is in line 65

Line 77- define “continuous basis”

Line 77-80- suggested rephrase: “Concentrates are hauled on a continuous basis year-round in 80 ton covered trucks from the Red Dog Mine Site to concentrate storage buildings at the Red Dog Port Site via the 84 km long Delong Mountain Transportation System haul road (DMTS, also known as the Red Dog haul road).”

Line 84- traverse instead of traverses?

Line 96- comma after S

Line 101- why less contamination expected?

Line 138-147- sentences emphasize/lead with authors instead of the results, could probably reduce paragraph length by switching the emphasis

Line 155- duplicated word “to to”

Line 223- give examples of sensitive nonvascular plants

Line 234-235- excellent check, thanks to the authors for stating explicitly

Line 266- any chance that toe slope has changed over time?

Line 279-280- define acrocarpous and pleurocarpous morphological. Explicate why these 5 groups were used

Line 282- of 4 x 8 plot as a whole or more fine spatial scale?

Line 283- 5 replicates where available? Ever less than that?

Line 292- “approximately 2 1 of the moss”? typo?

Line 293- what is a field duplicate?

Line 294- “reported” used twice in one sentence

Line 315-336- excellent and necessary explanations often omitted! Thanks to the authors for including!

Line 340-351- citation reference error

Line 352- why the testing in so many configurations? Is this fishing for results? Why not be more focused in approach? Please explicate. After reading late lines and explanations I think this introductory statement could be omitted or at least deemphasized.

Line 356- “empty”? is lacking lichen species the intention?

Line 392- define MRPP abbreviation

Line 396- why deleted species with 1 or fewer occurrences for lichens but 2 or fewer occurrences for vascular?

Line 465- how much did vigor vary within plots? It seems like specimens collection for lab work would likely trend toward being the most vigorous (sample bias)

Line 469- more *Hylocomium* sentence adjacent to previous *Hylocomium* sentence

Line 478- move Fig 3 reference to following sentence? Does not work well here.

Line 604-605- please explain why Fig 4 suggests this (i.e., spread along Axis 2?)

Line 664- “the upshot of this summarization” – too colloquial, rephrase

Line 681- ≥ 95 and ≤ 95 are both used while one should be < or >

Line 678-682- I think I understand what the authors are getting at but this paragraph is very confusing. The percentages don’t add up to 100 and the sign switches, consider simplifying or rephrasing.

Line 702-704- But the area within the easement north of the road is also closer to the road than the area south of the road

Line 731- the negative side of axis 1 is not apparent in Fig 8

Line 744- spell out “7”

Line 848- missing bracket

Line 855- replace “limited” with “limiting”

Line 880- refrain from use of “significant” non-mathematically

Line 889- this statement implies closure is imminent. Is it?

Line 892- subheading is confusing

Line 895, 897, 922…through 958- different format for citation reference with parentheses

Line 904-907- this phrasing of explanation is much clearer than in the results!

Line 925- Exponent, Inc.

Line 978- 4000 m for consistency

Line 1006- “CAKR’s lichen decline” suggests the effects are monument wide!

Line 1027-1033- some of this is already stated in the intro, consider omission or shortening

Line 1044- “natural number” could be replaced with “potential” or other word with less value associated

Line 1053- either in our out, replace or; also no clear explanation of the concept of “land status mixing”

Line 1060- add length of road

Line 1074- space before 75%

Line 1086-1087- rephrase to “following more intensive fugitive dust control or mine closure

Table and Figure Comments

Figure 2- I could not find reference to the “moss-only” plots in the text.

Figure 3- please increase resolution of axis ticks/labels and change x axis to display real/interpretable values, or in caption detail equivalent distances to axis values (similar to what authors did in S1Fig caption or better yet the axis used in S1File)

Figure 4- add sentence defining _2001 values like there is in Figure 9 caption

Table 1- “dw” means “dry weight”?

Figure 6- that confidence interval seems very very small…I suppose that could be because they are modeled values but perhaps worth a confirmation?

Figure 7- explicate the two inset maps in the caption. Presumably they are tied directly with the info in Table 5 but that is not indicated

Table 7- it is unclear whether pleurocarpous mosses includes *Hylocomium* here

S2 File- great addition to include this comparison
