# Supplementary material for: Impacts on tundra vegetation from heavy metal-enriched fugitive dust on National Park Service lands along the Red Dog Mine haul road, Alaska
Source: PLoS One. 2022 Jun 13;17(6):e0269801. doi: 10.1371/journal.pone.0269801 (PMC9191729; doi:10.1371/journal.pone.0269801)
Supplement: Attachment — Submitted filename: Response To Reviewers.docx [file pone.0269801.s010.docx]

From: Peter Neitlich, National Park Service

To: Dr. Tim Mousseau, Academic Editor, PLOS ONE

Re: Response to Reviewers

Date: May 16, 2022

Dear Dr. Mousseau,

Thank you for forwarding the excellent suggestions for revisions to this manuscript. These comments have helped tremendously in creating the polished version of the final manuscript. I have responded to each revision suggestion and have summarized resulting revisions in the comment matrix below. I am confident that we captured the essence of the requested revisions, but please do let me know if you still see any unresolved issues.

Best wishes,

Peter Neitlich

| **Reviewer** | **Comment** | **Revision** |
| --- | --- | --- |
| **Academic Editor** |  |  |
| Academic Editor | A rebuttal letter that responds to each point raised by the academic editor and reviewer(s). You should upload this letter as a separate file labeled 'Response to Reviewers'. | Current Document “Response to Reviewers.” |
| Academic Editor | A marked-up copy of your manuscript that highlights changes made to the original version. You should upload this as a separate file labeled 'Revised Manuscript with Track Changes'. | Attached. |
| Academic Editor | An unmarked version of your revised paper without tracked changes. You should upload this as a separate file labeled 'Manuscript'. | Attached. |
| Academic Editor | If you would like to make changes to your financial disclosure, please include your updated statement in your cover letter. |  |
| Academic Editor | If applicable, we recommend that you deposit your laboratory protocols in protocols.io to enhance the reproducibility of your results. | Protocols are cited as standard analytical laboratory protocols for elemental analysis. No novel techniques used. |
| Academic Editor | Please ensure that your manuscript meets PLOS ONE's style requirements, including those for file naming. | Done. |
| Academic Editor | Thank you for stating the following in the Acknowledgments Section of your manuscript:  "This research was funded by the National Park Service’s Arctic Network, Inventory and Monitoring Program and the Western Arctic National Parklands."  We note that you have provided funding information that is not currently declared in your Funding Statement. However, funding information should not appear in the Acknowledgments section or other areas of your manuscript. We will only publish funding information present in the Funding Statement section of the online submission form.  Please remove any funding-related text from the manuscript and let us know how you would like to update your Funding Statement. Currently, your Funding Statement reads as follows:  "This study was funded by the National Park Service Arctic Network and the Western Arctic National Parklands. The funders had no role in study design, data collection and analysis, decision to publish, or preparation of the manuscript."  Please include your amended statements within your cover letter; we will change the online submission form on your behalf. | Funding statement revised in cover letter. Thank you for that opportunity. |
| Academic Editor | 1. Please include your full ethics statement in the ‘Methods’ section of your manuscript file. In your statement, please include the full name of the IRB or ethics committee who approved or waived your study, as well as whether or not you obtained informed written or verbal consent. If consent was waived for your study, please include this information in your statement as well. | Ethics statement added, lines 179-183. |
| Academic Editor | 1. We note that Figures 1, 2, and 7 in your submission contain [map/satellite] images which may be copyrighted. All PLOS content is published under the Creative Commons Attribution License (CC BY 4.0), which means that the manuscript, images, and Supporting Information files will be freely available online, and any third party is permitted to access, download, copy, distribute, and use these materials in any way, even commercially, with proper attribution. For these reasons, we cannot publish previously copyrighted maps or satellite images created using proprietary data, such as Google software (Google Maps, Street View, and Earth). For more information, see our copyright guidelines: <http://journals.plos.org/plosone/s/licenses-> and-copyright.   We require you to either (1) present written permission from the copyright holder to publish these figures specifically under the CC BY 4.0 license, or (2) remove the figures from your submission:   a. You may seek permission from the original copyright holder of Figures 1, 2, and 7 to publish the content specifically under the CC BY 4.0 license.   We recommend that you contact the original copyright holder with the Content Permission Form [(http://journals.plos.org/plosone/s/file?id=7c09/content-permission-form.pdf)](http://journals.plos.org/plosone/s/file?id=7c09/content-permission-form.pdf)) and the following text:  “I request permission for the open-access journal PLOS ONE to publish XXX under the Creative Commons Attribution License (CCAL) CC BY 4.0 [(http://creativecommons.org/licenses/by/4.0/).](http://creativecommons.org/licenses/by/4.0/)) Please be aware that this license allows unrestricted use and distribution, even commercially, by third parties. Please reply and provide explicit written permission to publish XXX under a CC BY license and complete the attached form.”  Please upload the completed Content Permission Form or other proof of granted permissions as an "Other" file with your submission.  In the figure caption of the copyrighted figure, please include the following text: “Reprinted from [ref] under a CC BY license, with permission from [name of publisher], original copyright [original copyright year].”   b. If you are unable to obtain permission from the original copyright holder to publish these figures under the CC BY 4.0 license or if the copyright holder’s requirements are incompatible with the CC BY 4.0 license, please either i) remove the figure or ii) supply a replacement figure that complies with the CC BY 4.0 license. Please check copyright information on all replacement figures and update the figure caption with source information. If applicable, please specify in the figure caption text when a figure is similar but not identical to the original image and is therefore for illustrative purposes only. | The hillshade map presented in Figs 1, 2, 7 were created from the USGS National Elevation Dataset. As stated in the attached document USGS NED, all USGS National Elevation Dataset data are public domain and may be used freely without copyright permission. Documentation attached. |
| Academic Editor | Please review your reference list to ensure that it is complete and correct. If you have cited papers that have been retracted, please include the rationale for doing so in the manuscript text, or remove these references and replace them with relevant current references. Any changes to the reference list should be mentioned in the rebuttal letter that accompanies your revised manuscript. If you need to cite a retracted article, indicate the article’s retracted status in the References list and also include a citation and full reference for the retraction notice. | Completed. |
|  |  |  |
| **Reviewer #1** |  |  |
| Reviewer #1 | Search for references of “the Monument” and standardize capitalization | We have capitalized monument only as “Cape Krusenstern National Monument”. Otherwise it will be referred to generically as “monument” as is customary for parks and monuments. One change made. |
| Reviewer #1 | Standardize use of metric or miles throughout or include both | Both references to miles changed to km. One was a mistake (thanks for catching that), and the other colloquial use, changed. |
| Reviewer #1 | Standardize use of “haul road”, perhaps when first introducing the DMTS more formal name state “hereafter referred to as…” | Change made, standardized as suggested. DMTS retained only at beginning, in Figure captions and Table legends. |
| Reviewer #1 | Standardize use of elemental names and abbreviations and abbreviation definitions throughout | Standardized in name and order, thank you. |
| Reviewer #1 | Consider the use of subheadings in the Introduction if permitted by journal to facilitate comprehensive of the multiple issues (i.e., land ownership, history of deposition, use of nonvascular plants as monitors) | Considered, but could not figure out how to best use subheadings as the text ranges widely between topics. |
| Reviewer #1 | Nonconventional referencing to citation 17, the preceding study in Plos One, in the Methods and Abstract. Perhaps make more explicit statement of how this article is a continuation/extension of that study? | Done: “The current study is a continuation of the elemental concentration modeling in Neitlich et al. [17] and is based on the same field work.” |
| Reviewer #1 | Because the reference plots were later omitted, I think the authors can take out any reference to them in methods or results. Given the number of plots, I don’t see a real need for even stating that the authors did them even though it was a noble idea. Alternatively, since they are included in the discussion, make clear in the results why they still retain value in environmental comparison, even if they don’t for diversity comparison. | Clarified: “Due to vegetation type mismatch, the reference plots were not useable for diversity comparisions, but were retained for other comparisons of environmental co-variates and metrics of nonvascular plant vigor below.”  More generally, I think it’s important to keep the reference plots in, because many readers would otherwise wonder why we didn’t try to establish reference plots. |
| Reviewer #1 | Line 33- end with “within CAKR” instead of “in the monument”- monument is a bit ambiguous | Accepted. |
| Reviewer #1 | Line 44-47- confusing sentence expressing both negative and positive correlations | Leaving as is. |
| Reviewer #1 | Line 48-49- Wow, nicely put. It is easy to envision this impact. | Thanks. |
| Reviewer #1 | Line 67- the first reference of the NPS acronym is in line 65 | Changed, thanks. |
| Reviewer #1 | Line 77- define “continuous basis” | Rephrased as proposed. |
| Reviewer #1 | Line 77-80- suggested rephrase: “Concentrates are hauled on a continuous basis year-round in 80 ton covered trucks from the Red Dog Mine Site to concentrate storage | Accepted. |
| Reviewer #1 | Line 84- traverse instead of traverses? | Staying with singular rather than plural form: the road “traverses” |
| Reviewer #1 | Line 96- comma after S | Accepted. |
| Reviewer #1 | Line 101- why less contamination expected? | Clarified. |
| Reviewer #1 | Line 138-147- sentences emphasize/lead with authors instead of the results, could probably reduce paragraph length by switching the emphasis | Accepted and changed. |
| Reviewer #1 | Line 155- duplicated word “to to” | Accepted. |
| Reviewer #1 | Line 223- give examples of sensitive nonvascular plants | Clarified to lichens and bryophytes, with citations given. |
| Reviewer #1 | Line 234-235- excellent check, thanks to the authors for stating explicitly |  |
| Reviewer #1 | Line 266- any chance that toe slope has changed over time? | The road bed angles are quite steep so the distances to the truck lanes are likely to have stayed fairly constant. The plots are permanently marked for remeasurement accuracy. No change made. |
| Reviewer #1 | Line 279-280- define acrocarpous and pleurocarpous morphological. Explicate why these 5 groups were used | Defined and explicated. |
| Reviewer #1 | Line 282- of 4 x 8 plot as a whole or more fine spatial scale? | Changed to “plot” |
| Reviewer #1 | Line 283- 5 replicates where available? Ever less than that? | Changed to include “where available” |
| Reviewer #1 | Line 292- “approximately 2 1 of the moss”? typo? | “l” is for liters, no typo. I would like to say “liters” , but this is less standard as I use m, km, cm throughout the manuscript. |
| Reviewer #1 | Line 293- what is a field duplicate? | Defined. |
| Reviewer #1 | Line 294- “reported” used twice in one sentence | Changed to “described”. |
| Reviewer #1 | Line 315-336- excellent and necessary explanations often omitted! Thanks to the authors for including! |  |
| Reviewer #1 | Line 340-351- citation reference error | Fixed |
| Reviewer #1 | Line 352- why the testing in so many configurations? Is this fishing for results? Why not be more focused in approach? Please explicate. After reading late lines and explanations I think this introductory statement could be omitted or at least deemphasized. | This is a normal approach in community ecology. Data is tested in a variety of configurations to find the best fit, lowest stress, highest orthogonality, lowest skew, and best correlations with covariates. Explanation provided in text. |
| Reviewer #1 | Line 356- “empty”? is lacking lichen species the intention? | Clarified. |
| Reviewer #1 | Line 392- define MRPP abbreviation | Defined above. |
| Reviewer #1 | Line 396- why deleted species with 1 or fewer occurrences for lichens but 2 or fewer occurrences for vascular? | Deleting 2 species optimized the fit better than deleting 1, which had been sufficient for the lichen data. Inserted clarifying text. |
| Reviewer #1 | Line 465- how much did vigor vary within plots? It seems like specimens collection for lab work would likely trend toward being the most vigorous (sample bias) | Clarified to read that “*Hylocomium* measurements were obtained on 10 randomly selected individuals within each plot’s target collection of 2 liters of that species.” |
| Reviewer #1 | Line 469- more *Hylocomium* sentence adjacent to previous *Hylocomium* sentence | Accepted. |
| Reviewer #1 | Line 478- move Fig 3 reference to following sentence? Does not work well here. | Accepted. |
| Reviewer #1 | Line 604-605- please explain why Fig 4 suggests this (i.e., spread along Axis 2?) | Clarified as follows: “The wide spread of plots in the 10 m and 100 m distances classes on Axis 2 (Fig 4) suggests that this axis may have just explained random species occurrence or survivorship in a zone with few species.” |
| Reviewer #1 | Line 664- “the upshot of this summarization” – too colloquial, rephrase | Changed as follows: “In short, the model summarization showed a 100% probability of impacts to LSR out to 3000 m and very high probability of impacts out to 4000 m.” |
| Reviewer #1 | Line 681- ≥ 95 and ≤ 95 are both used while one should be < or > | Changed to >95%. |
| Reviewer #1 | Line 678-682- I think I understand what the authors are getting at but this paragraph is very confusing. The percentages don’t add up to 100 and the sign switches, consider simplifying or rephrasing. | Good suggestion. I think I significantly clarified it and added a percentages column to Table 6 to make it even more clear. |
| Reviewer #1 | Line 702-704- But the area within the easement north of the road is also closer to the road than the area south of the road | Good point. Clarified. |
| Reviewer #1 | Line 731- the negative side of axis 1 is not apparent in Fig 8 | Disagree. Everything to the left of the centroid mark is negative. |
| Reviewer #1 | Line 744- spell out “7” | Accepted. |
| Reviewer #1 | Line 848- missing bracket | Accepted. |
| Reviewer #1 | Line 855- replace “limited” with “limiting” | Accepted. |
| Reviewer #1 | Line 880- refrain from use of “significant” non-mathematically | Changed to “key”. |
| Reviewer #1 | Line 889- this statement implies closure is imminent. Is it? | Clarified. |
| Reviewer #1 | Line 892- subheading is confusing | Changed to “Estimating Background LSR” |
| Reviewer #1 | Line 895, 897, 922…through 958- different format for citation reference with parentheses | Corrected. |
| Reviewer #1 | Line 904-907- this phrasing of explanation is much clearer than in the results! | I attempted to clarify additionally. |
| Reviewer #1 | Line 925- Exponent, Inc. | Corrected. |
| Reviewer #1 | Line 978- 4000 m for consistency | Accepted. |
| Reviewer #1 | Line 1006- “CAKR’s lichen decline” suggests the effects are monument wide! | Changed to “the reported lichen decline” rather than “CAKR’s lichen decline”. |
| Reviewer #1 | Line 1027-1033- some of this is already stated in the intro, consider omission or shortening | Deleted repeating lines. |
| Reviewer #1 | Line 1044- “natural number” could be replaced with “potential” or other word with less value associated | Changed to potential number. |
| Reviewer #1 | Line 1053- either in our out, replace or; also no clear explanation of the concept of “land status mixing” | Changed and clarified: “the joint presence of grid cells attributed as either inside or outside of the easement” |
| Reviewer #1 | Line 1060- add length of road | Accepted. |
| Reviewer #1 | Line 1074- space before 75% | Accepted. |
| Reviewer #1 | Line 1086-1087- rephrase to “following more intensive fugitive dust control or mine closure | Accepted. |
| Reviewer #1 | Figure 2- I could not find reference to the “moss-only” plots in the text. | Good catch. The “moss-only” plots were from Neitlich et al. 2017 PLOS publication on spatial patterns of metals deposition. They are not relevant to this study and were removed from the figure. |
| Reviewer #1 | Figure 3- please increase resolution of axis ticks/labels and change x axis to display real/interpretable values, or in caption detail equivalent distances to axis values (similar to what authors did in S1Fig caption or better yet the axis used in S1File) | Figure axes changed to match S1 File. |
| Reviewer #1 | Figure 4- add sentence defining _2001 values like there is in Figure 9 caption Table 1- “dw” means “dry weight”? | Added to Fig 4 and Fig 9 captions. |
| Reviewer #1 | Figure 6- that confidence interval seems very very small…I suppose that could be because they are modeled values but perhaps worth a confirmation? | These are indeed the 95% CI. |
| Reviewer #1 | Figure 7- explicate the two inset maps in the caption. Presumably they are tied directly with the info in Table 5 but that is not indicated | Clarified in added text: “The upper and lower bounds of the 95% confidence interval (i.e., 97.5 and 2.5 percentile values of the 200 simulations) are provided in small maps below the main map.” |
| Reviewer #1 | Table 7- it is unclear whether pleurocarpous mosses includes *Hylocomium* here | Sentence added: “*Hylocomium splendens* does not appear as all indicator values for this group had p values > 0.05.” |
| Reviewer #1 | S2 File- great addition to include this comparison | Thanks. |
|  |  |  |
| **Reviewer #2** |  |  |
| Reviewer #2 | This title is very wordy, I suggest simplifing the title. Impacts of heavy metals along the Red Dog road in Alaska.  The current title focuses too much on lichens and not on the other taxa and many readers interested in vascular plants might not bother with an article with the current title. | Changed to: Impacts on tundra vegetation from heavy metal-enriched fugitive dust on National Park Service lands along the Red Dog Mine haul road, Alaska. |
| Reviewer #2 | The differences in response to fugitive dust contaminants among the different vegetation components  170 (i.e., lichens, bryophytes, vascular plants) is unique for this study and should be of interest to a very wide audience. | Agree. |
| Reviewer #2 | In the Taxonomy section of the Methods. It would be clearer to state;  Specimens of known genus and unknown species—and for which all species likely to occur at 335 the site were already present—were lumped with the most common species in the data set. 336 Examples of this included "species with in the" Sphaerophorus and Bryoria :genera: | Clarified. |
| Reviewer #2 | 559 species in plots close to the road was visible to observers and in photos (Fig 5). While most 560 lichens species were eliminated at distances close to the road (10–50 m), those present included: 561 (> 50% frequency) Thamnolia subuliformis, Flavocetraria cucullata, Cladonia amaurocraea, 562 and (33–50% frequency) Cladonia stygia, Cladonia arbuscula, Cetraria laevigata, Cladonia 563 maxima, Cladonia rangiferina, Peltigera aphthosa. These are presumably the most pollutant5   Given the data set it seems that there should be a qualifier of what type of pollutant these lichens species are tolerant of? not all pollutants? just these heavy metals? | Excellent point. Changed to: “These are presumably the most tolerant species in the study to pollutants potentially including Zn, Pb, Cd, and sulfides.” |
